# Tunnel ultrasound can guide the use of peritoneal dialysis catheter exit site relocation by external splicing and cuff removal in refractory tunnel infection

**DOI:** 10.1007/s11255-024-04023-7

**Published:** 2024-03-20

**Authors:** Luca Nardelli, Antonio Scalamogna, Federica Tripodi, Chiara De Liso, Carlo Alfieri, Giuseppe Castellano

**Affiliations:** 1 Division of Nephrology, Dialysis and Kidney Transplantation, Fondazione IRCCS Ca’ Granda Ospedale Maggiore Policlinico, Via della Commenda 15 20122, Milan, Italy; 2 Department of Clinical Sciences and Community Health, Università degli studi di Milano, Milan, Italy

**Keywords:** Peritoneal dialysis, Exit-site infection, Tunnel infection, Peritonitis, Ultrasonography, Ultrasound, Cuff shaving, Cuff removal, Partial reimplantation, Splicing

## Abstract

**Background:**

Peritoneal dialysis (PD) catheter related infections continue to be a major cause of morbidity and transfer to hemodialysis (HD) in PD patients. The treatment of tunnel infection (TI) could be challenging, especially when the infection involves the superficial cuff requiring the removal of the catheter. To spare the patient the loss of the catheter and the transfer to HD, several mini-invasive surgical techniques have been proposed as rescue therapy. Furthermore, nowadays, the rapid growth of digital technology has enormously increased the diagnostic sensibility of the echo signal allowing to accurately defines the extent of the infectious process along the PD catheter tunnel.

**Methods:**

Between 1st January 2020 and 31st December 2021 seven patients who underwent exit-site relocation by external splicing and cuff removal at our institution due to refractory TI were included in the study. All patients were followed until 12 months after the procedure. As soon as TI was defined refractory to the medical therapy, an ultrasonographic examination of the catheter tunnel was performed to define the extent of the infectious episode.

**Results:**

Among the 7 infectious episodes, 4 were caused by *P. aeruginosa*, and 3 by *S. aureus*. Around the superficial cuff the hypo/anechoic collections detected by ultrasounds showed a mean diameter of 3.05 ± 0.79 mm. The exit-site relocation by external splicing and cuff removal was successful in all cases (7/7, 100%).

**Conclusions:**

In our experience the use of exit site relocation by external splicing and cuff removal as rescue therapy for TI with positive ultrasounds for TI limited to superficial cuff involvement and without secondary peritonitis, yielded to promising results with a success rate of 100%. This preliminary experience underlines the paramount usefulness of tunnel echography in accurately defining the extent of TI and, consequently, guiding the choice of the therapeutical approach in refractory TI.

## Introduction

Peritoneal dialysis (PD) catheter related infections can lead to peritonitis, hospitalization, shift to hemodialysis (HD) and death [1–3]. Nearly 20% of the exit site infections may arise in conjunction with or develop into tunnel infection (TI), while up to 60% of TI are refractory to medical therapy requiring the removal of the PD catheter [4].

In fact, the spread of the infective process to the superficial cuff makes the infection hardly responsive to the antibiotic therapy since the microorganisms can infiltrate the Dacron of the catheter cuffs and form a biofilm in this area that facilitates their proliferation [4, 5]. The creation of this layer becomes a barrier for antibiotic agents [6, 7]. As a consequence, TI with involvement of the cuffs is unlikely to respond to antibiotic therapy, requiring often the removal of the catheter [1, 8–10]. However, this management necessitates the interruption of PD and the transition to HD via the placement of a temporary central venous catheter.

Conversely, the optimal therapeutic approach should allow continuation of PD with an uninfected catheter. Thus, several noninvasive surgical techniques, such as the cuff-shaving, the partial reimplantation of the PD catheter or the exit-site relocation by external splicing and superficial cuff removal have been suggested [11, 12].

Furthermore, the rapid growth of digital technology has generated a solid background for the rapid development of ultrasonographic techniques allowing an increasingly refined analysis of the diagnostic information of the echo signal [13]. Thus, nowadays the ultrasound evaluation of the PD catheter tunnel represents a non-invasive, easily tolerated and readily available method with high sensitivity in experienced hands [14]. However, pre-operative ultrasonographical examination has never been used as a tool to select the more appropriate approach in case of refractory TI. Recently, our group has proposed a specific algorithm that, combining anatomical and ultrasonographical features, can guide the treatment choice [11, 13]. Thus, in order to obtain preliminary data that could initially validate this approach and corroborate the ultrasonographical diagnostic criteria, we exploited part of this algorithm to treat seven cases of refractory TI by external splicing and cuff removal procedure.

## Methods

### Participants and study design

This is a single center retrospective cohort study carried out at Fondazione IRCCS Ca’ Granda Ospedale Maggiore Policlinico between January 1st 2020 and December 31st 2021. Adults (> 18 years) who showed a TI resistant to antibiotic therapy and positive ultrasonographic findings of superficial cuff involvement with no signs of infection beyond this point, absence of secondary peritonitis, and exit-site/superficial cuff distance greater than 2 cm, were included in the study. Those patients were candidate for cuff removal and were followed until 12 months after the intervention. The data were collected by reviewing an electronic database (Galenus^®^, Infogramma s.r.l., Milan, Italy).

### Clinical management

In every patient a straight double-cuffed Tenckhoff catheter was placed with a modified double purses string technique around the inner-cuff either in semi-surgical or surgical procedure, as described elsewhere [15, 16]. The creation of the subcutaneous part was achieved by using a piercing tunneller in a direction able to minimize shear forces with the superficial cuff located at least 4 cm from the exit of the skin [4]. As far as routinely exit-site care is concerned, patients were instructed to apply hydrogen peroxide followed by 5% sodium hypochlorite solution to the skin surface three to four times per week.

Tunnel infection was diagnosed in the presence of clinical inflammation (erythema, swelling, tenderness or induration) along the catheter tunnel [1].

Empirical oral antibiotic with cephalexin 500 mg bid was initiated in all patients at the clinical diagnosis of TI. Subsequently, the antibiotic therapy was adjusted according to the antibiogram results. In the case of methicillin-sensitive *S. aureus* cephalexin was continued. Differently, when *P. aeruginosa* was isolated, the patients were shifted to oral ciprofloxacin 250 mg bid while if methicillin-resistant *S. aureus* was identified, they were treated with intravenous vancomycin associated to oral rifampicin 300 mg bid.

**Fig. 1:**
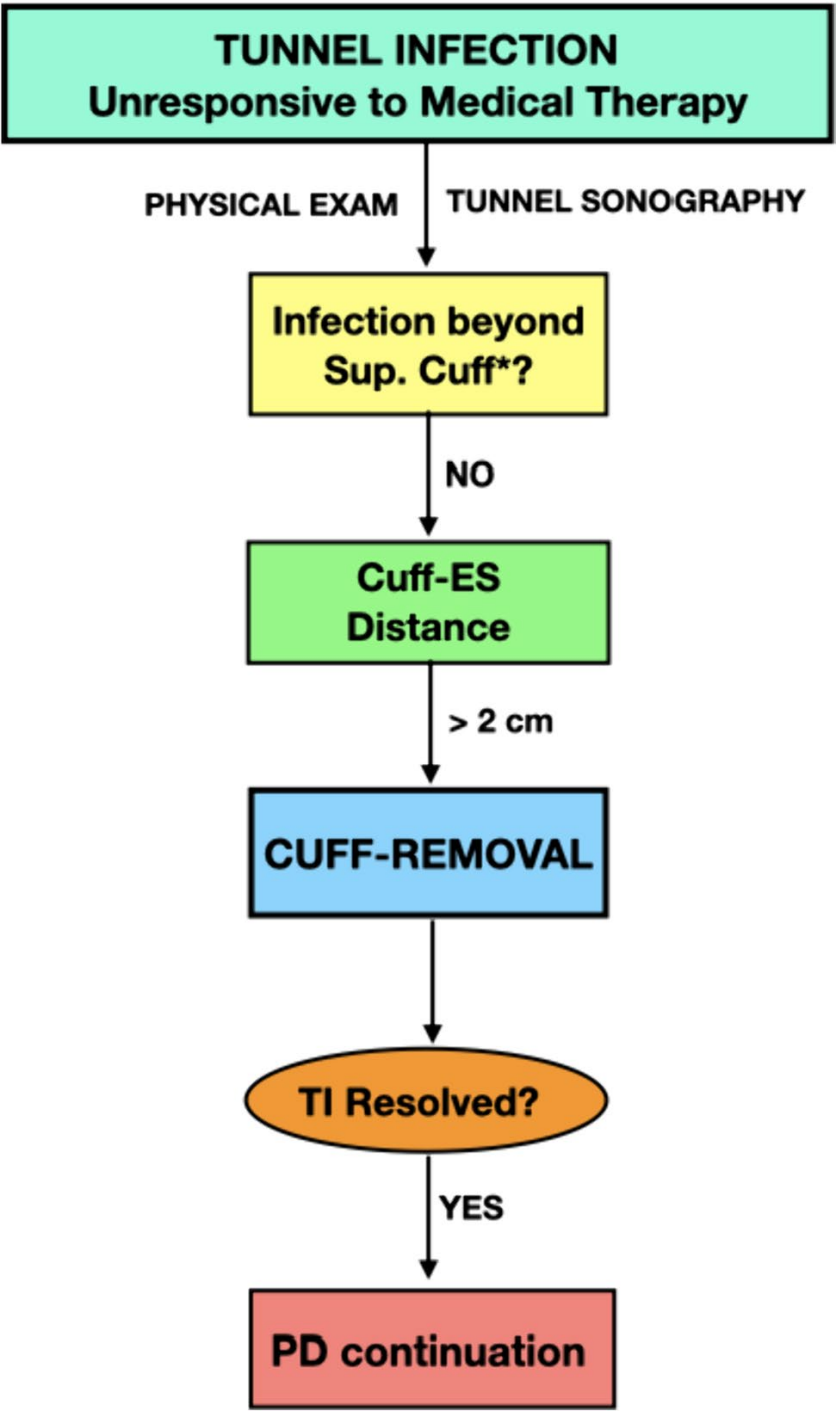
Flowchart regarding the management of tunnel infection unresponsive to medical therapy with concomitant superficial cuff infection, but without ultrasonographic evidence of infection spreading beyond the superficial cuff and an exit-site/superficial cuff distance greater than 2 cm. *CUFF-REMOVAL* exit-site relocation by external splicing and cuff removal, *ES * exit-site, *PD* peritoneal dialysis, *Sup.* *Cuff* superficial cuff, *TI* tunnel infection. *The extent of the infectious process should be assessed by physical exam (searching for erythema, swelling, induration, or tenderness over the subcutaneous pathway) and by ultrasonographic evaluation (detection of hypoechogenic area between the tube/cuff of the catheter and the surrounding tissues with a diameter > 2 mm) [modified by [11]]

All the episodes were defined refractory to the medical therapy in case of no improvement after two weeks of targeted antibiotic treatment or 3-weeks for TI due to *P. aeruginosa* [1]. At this time, a careful ultrasound examination of the catheter tunnel was performed. Then, provided a positive ultrasonographic findings of superficial cuff involvement (hypo/anechoic area with a diameter > 2 mm), but no signs of infection beyond this point as well as absence of secondary peritonitis, exit-site relocation by external splicing and cuff removal was employed as a catheter rescue therapy in case of an exit-site/superficial cuff distance greater than 2 cm (Fig. 1). After the surgical procedure, the antibiotic treatment was continued for further 3 weeks.

### Surgical procedure

**Fig. 2:**
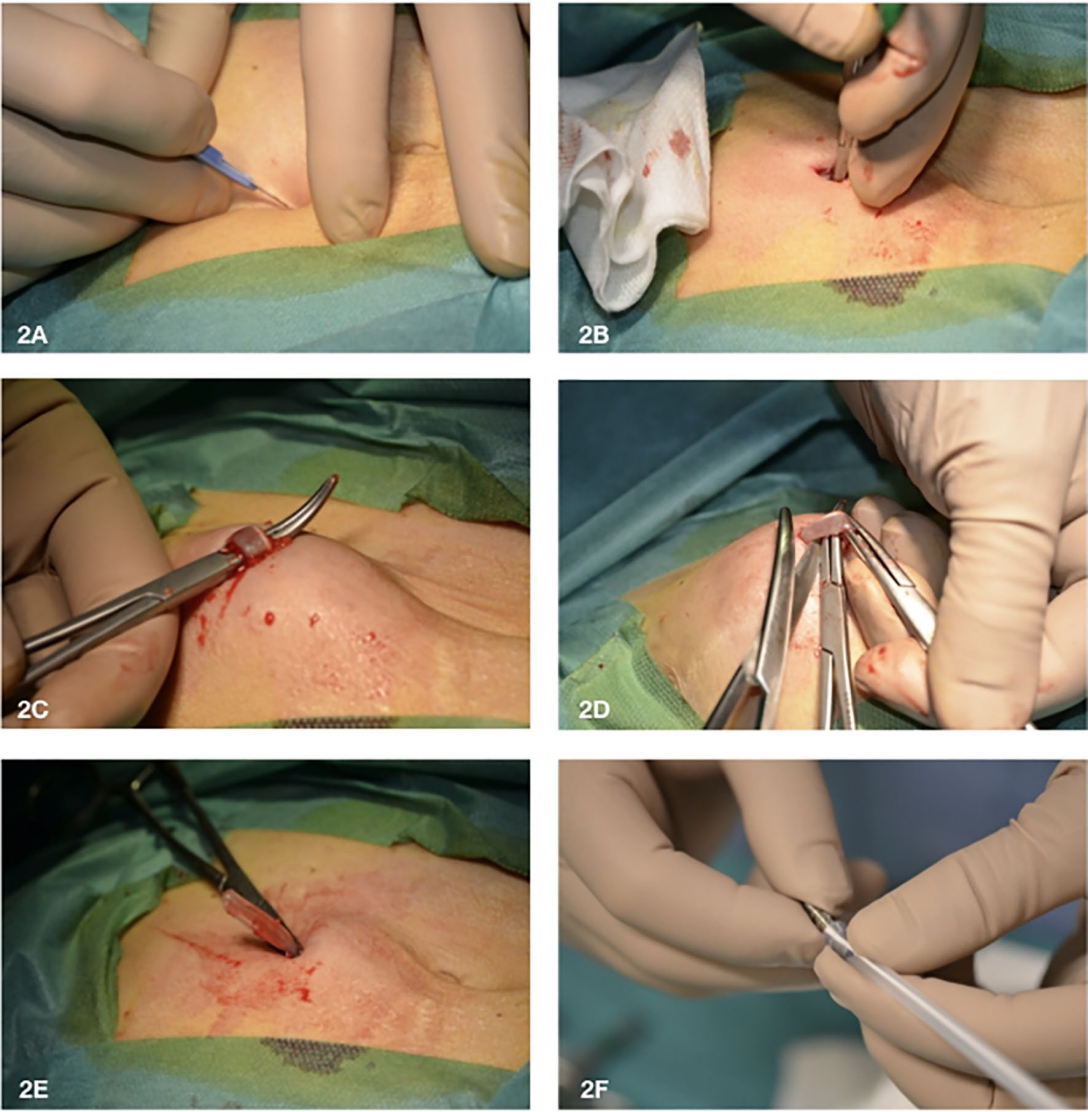
**A**–**F** Exit-site relocation by external splicing and cuff removal—first part. **A** Skin incision of approximately 5 mm at 1–2 cm distance from the superficial cuff in the direction of the deep cuff; **B** through the tiny opening the peritoneal catheter is retrieved from the subcutaneous tissue; **C** then it is lifted out from the skin; **D** and is cut 1 cm distance from the superficial cuff; **E** now the free end of the catheter is dragged out from the s subcutaneous layer; **F** subsequently, part of a new catheter is taken and one of the two free ends is attached to a double-barbed titanium extender

**Fig. 3:**
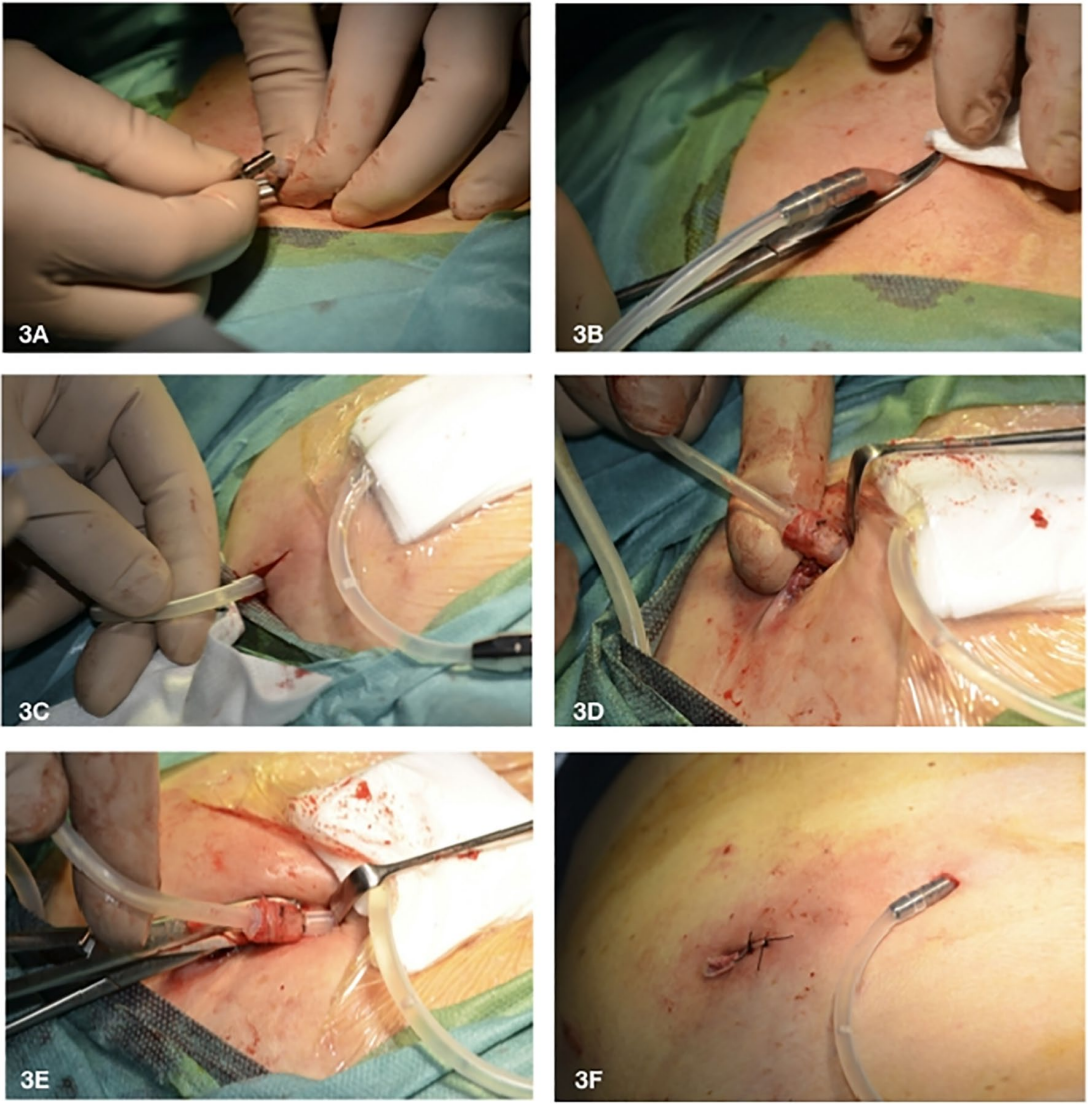
**A**–**F** Exit-site relocation by external splicing and cuff removal—second part. **A** the catheter is externally lengthened by connecting its remaining free end to the second edge of the double-barbed titanium extender; **B** the adapter used to extend the catheter remains outside the new exit-site to not hinder its maturation; **C** skin incision of about 1–2 cm at the level of the old exit-site sinus is carried out; **D** then, the adipose and scar tissue adjacent to the infected superficial cuff is removed; **E** eventually, the infected superficial cuff connected to the distal part of the old catheter is taken out through the old exit-site; **F** the new-exit site is distant 4–5 cm from the skin incision performed to remove the old exit-site

The exit-site relocation by external splicing and cuff removal consists in creating a skin incision of approximately 5 mm at 1–2 cm distance from the superficial cuff along the subcutaneous catheter course towards the deep cuff (Fig. 2A). Then, through this tiny opening, the peritoneal catheter is retrieved by a blunt dissection (Fig. 2B), lifted out from the skin (Fig. 2C), and cut 1 cm distance from the superficial cuff (Fig. 2D). After dragging out the free end of the catheter from the skin (Fig. 2E), it is externally lengthened by connecting a double-barbed titanium extender that was previously attached to new catheter piece (Figs. 2F, 3A, B). Hence, a skin incision of 2 cm is performed in proximity of the infected superficial cuff which is, then, isolated by a dissection of the surrounding tissue and, eventually, removed through the old exit-site (Fig. 3C–F) [11, 12]. The superficial cuff was conserved and sent for microbiological evaluation.

### Ultrasound examination

**Fig. 4:**
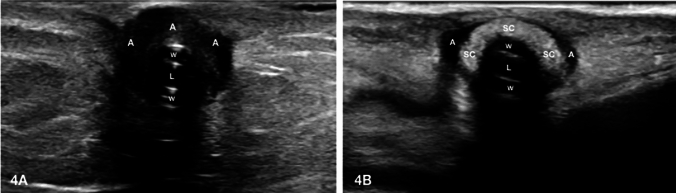
**A**, **B** Positive ultrasonographic findings of tunnel infection with superficial cuff involvement. **A** Presence of hypo/anechoic area with a diameter > 2 mm located between the catheter walls and the surrounding tissues; **B** Presence of hypo/anechoic area with a diameter > 2 mm around the hyperechoic signal of the Dacron cuff indicating the involvement of the superficial by the infectious process. *A* hypo/anechoic area (abscess), *L* lumen of the catheter, *SC* superficial cuff, *W* catheter walls

Before starting the ultrasonographic examination, the exit-site was carefully disinfected by applying hydrogen peroxide followed by 5% sodium hypochlorite solution and covered with a transparent sterile film dressing to avoid its contamination. Then, using a 7–13 MHz frequency linear probe (Affiniti 70G, Philips^®^, Amsterdam, Netherlands), the tunnel of the catheter was evaluated maintaining the patient in supine decubitus. Once the catheter was identified, it was followed, by both long and short-axis visualization, along the whole subcutaneous path from the exit-site until the peritoneal cavity. The examination was aimed at detecting the presence of pathological hypo/anechoic collection surrounding either the catheter walls or the Dacron cuffs. A hypo/anechoic area with a diameter > 2 mm located between the catheter walls and the surrounding tissues was acknowledged as a positive exam (Fig. 4A). The involvement of either the superficial or deep cuff by the infectious process was defined as the detection of the previous ultrasonographic criteria around the hyperechoic signal of the Dacron cuff (Fig. 4B).

### Statistical analysis

Normally distributed variables are presented as mean ± standard deviation, while nonparametric data are presented as median with interquartile range. Categorical variables are expressed as frequency and percentage.

## Results

### Population characteristics and causative organisms

The demographic and clinical details of the 7 participants are shown in Table 1. Most patients were male (57.1%) with a mean age of 63.7 ± 11.3 (standard deviation [SD]) years. The median time of continuous ambulatory PD treatment at TI diagnosis was 15.7 months (interquartile range 7.9–21.8). Among the 7 episodes of TI, 4 were caused by *Pseudomonas aeruginosa* (*P. aeruginosa*), while 3 by *Staphylococcus aureus* (*S. aureus*).

**Table 1:** Baseline characteristics and clinical details regarding the seven patients who underwent exit site relocation by external splicing and cuff removal as rescue catheter treatment for refractory tunnel infection

	ID-1	ID-2	ID-3	ID-4	ID-5	ID-6	ID-7
Sex (M = male; F = female)	F	M	F	M	F	M	M
Age (years)	49	69	81	70	54	55	68
Kidney disease	ADPKD	Unknown	Unknown	Diabetic Neph	Cardiorenal type II	IgA Neph	Membranous Neph
Time on PD (months)	11.4	4.3	17.4	26.2	3.2	15.7	34.4
PRE ATB Therapy	Cepha– > cipro	Cefa– > vanco	Cepha– > cipro	Cephalexin	Cepha– > cipro	Cephalexin	Cepha– > cipro
Microorganism	*P. aeruginosa*	*S. aureus*	*P. aeruginosa*	*S. aureus*	*P. aeruginosa*	*S. aureus*	*P. aeruginosa*
DUR PRE ATB therapy (days)	28	21	25	19	27	18	24
DIM AREA SUP CUFF (mm)	2.22	2.45	2.31	3.66	2.82	3.81	4.11
MAX DIM AREA PERICAT (mm)	3.24	3.64	3.43	7.82	3.55	4.13	5.43
POST ATB THERAPY	Ciprofloxacin	Vancomycin	Ciprofloxacin	Cephalexin	Ciprofloxacin	Cephalexin	Ciprofloxacin
DUR POST ATB THERAPY (days)	21	21	21	21	21	21	21
Relapse at 12 months	No	No	No	No	No	No	No

*ADPKD*  autosomal dominant polycystic kidney disease, *cepha* cephalexin, *cipro* ciprofloxacin, *DIM* dimension, *DUR* duration, *Neph* nephropathy, *ID* patient identification number, *PRE ATB THERAPY* antibiotic therapy prescribed before exit site relocation by external splicing and cuff removal (– >  = shift to other molecule during the antibiotic course), *POST ATB THERAPY* antibiotic therapy prescribed after the exit site relocation by external splicing and cuff removal, *PD* peritoneal dialysis, *PERICAT* pericatheter, *vanco* vancomycin

### Clinical outcomes

Around the superficial cuff the hypo/anechoic collections detected by ultrasonographic examination showed a mean diameter of 3.05 mm ± SD 0.79 mm (Table 1). However, the greatest dimension of the peri-catheter liquid collection was always found in a portion between the exit-site and the superficial cuff (mean value 4.46 mm ± SD 1.65 mm; Table 1). No signs of infection beyond the superficial cuff either by physical or ultrasonographic examination were identified.

In every case, the microbiological specimen of the superficial cuff, collected during the operation, identified the presence of the same microorganisms which had been previously isolated from the purulent discharge of the exit-site.

The exit-site relocation by external splicing and cuff removal was successful in all cases (7/7, 100%). In fact, after 12 months from the surgical procedure, physical and ultrasonographic examination of the tunnel resulted negative as well as the microbiological swab performed both at the old and new exit-site (Table 1). No patient needed to interrupt the peritoneal exchanges.

## Discussion

The available evidence has shown that tunnel infections with concomitant involvement of the cuffs are unlikely to respond efficaciously to antibiotic therapy, thereby requiring in most cases the removal of the catheter [8–10]. However, via ultrasonographic examination is possible to accurately determine the extent of the infectious process and to identify with high sensibility any liquid collection around the PD catheter, despite specific ultrasonographical criteria have not been precisely established yet.

The presence of an abscess surrounding the cuff corresponds to the dissemination of the infectious process in the Dacron material; hence, we decided to employ as diagnostic criteria for a positive ultrasonographical exam the presence of hypo/anechoic area with a diameter > 2 mm located between the cuff and the adjacent tissue.

In order to spare the patient the loss of the peritoneal catheter and the change of dialysis modality, our group has recently proposed a specific approach for the treatment of refractory TI [11, 13]. Within this algorithm, provided the absence of infection beyond the superficial cuff as well as secondary peritonitis, we suggested to consider the distance between the exit-site and the superficial cuff. If this distance was less than or equal to 2 cm, cuff-shaving was proposed, otherwise (distance > 2 cm) it was suggested to relocate the exit-site by external splicing and cuff removal or by partial reimplantation of the catheter [11].

According to our algorithm, in the seven cases reported the distance between the exit-site and the superficial cuff was greater than 2 cm, thus the exit-site relocation by external splicing and cuff removal was employed to treat the episodes of refractory TI without secondary peritonitis due to *P. aeruginosa* or *S. aureus* (Fig. 1). The procedure was successful in all cases (7/7, 100%). Indeed, after 12 months from the surgical procedure we did not observe any relapsing episodes.

In a prior analysis with the use of cuff removal technique, we obtained a less satisfying success rate of approximately 70% (15 out of 21 cases vs 7/7 of this series) in both Gram-positive (12 out of 17 cases) and Gram-negative infections (3 out of 4 cases) [11]. This outcome discrepancy might be due to the absence of pre-operative ultrasonographical evaluation in the previous experience. In fact, the ultrasonographic examination of the tunnel has proved to be as specific as the clinical parameters, but with a considerably greater sensitivity, especially in case of TI involving the portion of the tunnel comprised between the superficial and deep cuff [9, 10, 17, 18].

Thus, at that time, the surgical procedure could have been also carried out in cases in which the infection had been already spread beyond the superficial cuff making the mini-invasive surgical approach ineffective.

Conversely, upon the new protocol we always performed a pre-procedure ultrasonographic examination to define the extent of the infectious process that, eventually, guided the choice of the surgical technique [13]. Therefore, the precise localization via ultrasounds of the infection process could reasonably explain the significative outcome improvement observed in this series. It is worth of mentioning that, to the best of our knowledge, this is the first study in which the type of surgical procedure was decided on the basis of the findings observed by a pre-operative ultrasonographic examination of the subcutaneous tunnel.

In comparison to the cuff-shaving, the exit-site relocation by external splicing and cuff removal has the advantage of being less traumatic [19]. In fact, it is performed through a small incision that corresponds to the location of the new exit-site. This procedure does not require the removal of the fatty tissue around the infected cuff, while also limiting bleeding and wound pain [11, 12]. Contrary to the partial reimplantation technique, a shorter incision is performed [20–23]. In addition, the catheter is extended outside the skin allowing a rapid diagnosis and a prompt resolution in case of disconnection [11, 12].

However, besides the extent of the infection (progression deeper than the superficial cuff or presence of concurrent peritonitis) further contraindications of our procedure might be represented either by a long distance between the catheter and the skin or by a poor exit-site location.

**Fig. 5:**
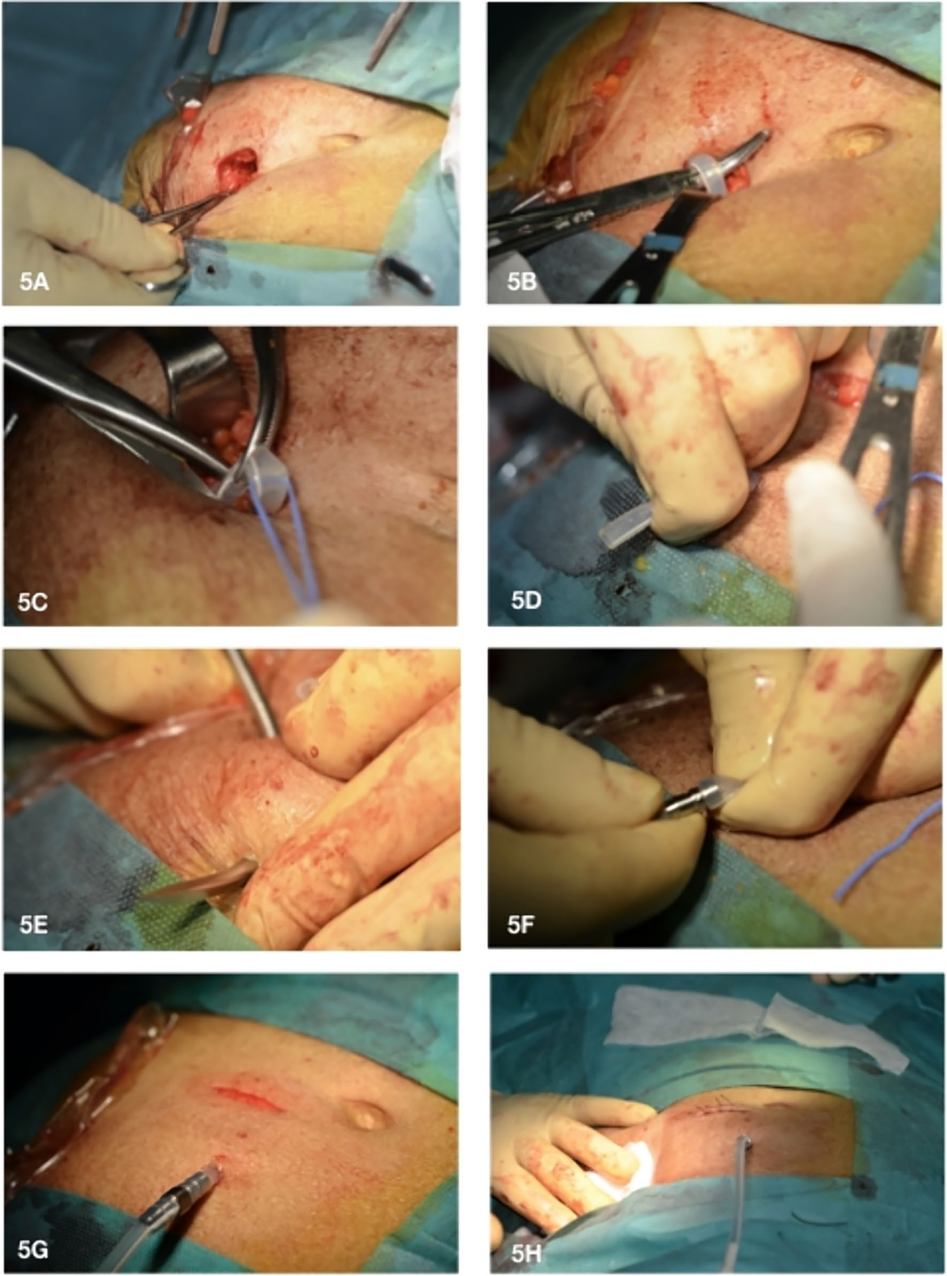
**A**–**H** Modified cuff removal technique by additional tunneling of the PD catheter. **A** skin incision of approximately 3–4 cm 1 at 1–2 cm distance from the superficial cuff along the catheter path in the direction of the deep cuff; **B** through the opening the peritoneal catheter is retrieved from the subcutaneous tissue **C** the catheter is cut 1 cm distance from the superficial cuff; **D** then, the free end of the catheter is lifted out from the skin; **E** subsequently, the catheter free end is attached to a metal bend rod and the isolated part of the catheter is dragged in the subcutis to create a new exit-site; **F-G** Once the catheter is taken out from the skin, it is externally lengthened by connecting a double-barbed titanium extender that was previously attached to new catheter piece; **H** now the new exit-site is located far away from the old one

In fact, in the first case the depth of the catheter complicates the isolation of the device trough a tiny cutaneous incision. While in the second case, the poor exit-site location constricts the creation of the new exit-site few centimeters from the old one (approximately a distance between the previous exit-site and the superficial cuff) often leading to unsatisfactory results. Thus, in these cases the partial reimplantation of the catheter may represent the elective technique. This procedure consists in isolating the portion of the catheter between the two cuffs and splicing it in proximity of the deep cuff. Thereafter, the device is extended via the portion of another catheter and tunneled subcutaneously in the contralateral hemi-abdomen with the creation of a new exit-site. Thus, this approach would allow to move the new exit-site far from the old one and to facilitate the isolation of the catheter since the skin incision is inevitably larger [11]. Another feasible option in the previous conditions (long distance between the catheter and the skin or a poor exit-site location) is represented by a modified technique of our procedure. The variation consists in carrying out a wider incision (approximately 3–4 cm) at 1–2 cm distance from the superficial cuff along the catheter path in the direction of the deep cuff that permits to isolate a longer portion of the subcutaneous device. Once the catheter is cut off at the proximity of the superficial cuff, the free end is attached to a metal bend rod and the isolated part of the catheter is dragged in the subcutis to create a new exit-site away from the old one. Eventually, once the catheter is taken out from the skin, it is externally lengthened by connecting the double-barbed titanium extender (Fig. 5A–H).

In conclusion, these preliminary results underline the usefulness of the integration of the physical examination with the ultrasound evaluation when deciding the therapeutic approach in the treatment of the refractory tunnel infection. In particular, in PD patients affected by TI with positive ultrasonographic findings of superficial cuff involvement (presence of hypo/anechoic area with a diameter > 2 mm between the cuff and the adjacent tissue), but no signs of infection beyond this point, and absence of secondary peritonitis, exit-site relocation by external splicing and cuff removal, as a rescue therapy for refractory TI, showed a success rate of 100%. Certainly, additional data collected on a larger population is needed to validate both the proposed ultrasonographical diagnostic criteria and the therapeutical algorithm.

## Data Availability

The data that support the findings of this study are not openly available, but are obtainable from the corresponding author upon reasonable request. Data are located in controlled access data storage at University of Milan.
